# Distinguishing artificial intelligence from robotic execution in total knee arthroplasty: a narrative review and evidence map

**DOI:** 10.1186/s42836-026-00444-0

**Published:** 2026-11-06

**Authors:** Muhammad Adil Malik, Song Wu, Wenxiu Zhang, Junjie Huang, Xu Cao

**Affiliations:** Department of Orthopedics, Central South University, The Third Xiangya Hospital, Changsha, 410013 China

**Keywords:** Total knee arthroplasty, Robotic-assisted surgery, Artificial intelligence, Machine learning, Osteoarthritis, Implant sizing, Outcome prediction, Patient selection, Intraoperative decision support, Evidence map

## Abstract

**Purpose:**

Artificial intelligence (AI) and machine-learning methods are increasingly discussed in relation to robotic-assisted total knee arthroplasty (TKA). However, robotic assistance, computer navigation, and AI-based prediction represent distinct functions that are often conflated under the single label “AI-assisted robotic TKA,” obscuring the state of evidence for each. This review clarifies these distinctions and maps the current evidence for AI-related functions across the TKA pathway, keeping AI prediction evidence analytically separate from robotic execution evidence at the level of each clinical function.

**Methods:**

We performed a critical narrative review and evidence map of peer-reviewed literature relevant to robotic-assisted TKA, navigation-assisted TKA, AI/ML prediction models, digital planning, implant sizing, intraoperative decision support, postoperative monitoring, implementation, and economic evaluation. Evidence was interpreted according to clinical function, comparator structure, validation status, and translational maturity. Because the available literature is methodologically and conceptually heterogeneous, quantitative pooling and formal comparative-effectiveness claims were not attempted.

**Results:**

Robotic-assisted and navigation-assisted TKA have the most mature evidence base for improving technical precision, particularly implant positioning and radiographic alignment. In contrast, AI/ML evidence is concentrated mainly in adjacent pathway functions, including radiographic interpretation, implant-sizing prediction, patient-selection support, dissatisfaction prediction, outcome modelling, perioperative risk stratification, and postoperative monitoring. Direct clinical evidence demonstrating that AI-enabled robotic TKA provides incremental patient-level benefit beyond conventional robotic assistance remains limited. Current studies are constrained by inconsistent terminology, short follow-up, limited external validation, incomplete reporting of AI components, heterogeneous outcomes, cost uncertainty, and uncertain generalizability across patient populations and healthcare settings.

**Conclusion:**

AI should not currently be treated as a single validated surgical intervention in TKA. Rather, it represents a family of emerging pathway-level functions that may support preoperative planning, intraoperative decision support, patient selection, risk communication, and longitudinal recovery monitoring. Future studies should explicitly define the AI component being evaluated, distinguish AI functionality from robotic or navigation assistance, use standardized comparators, and assess long-term patient-reported outcomes, implant survivorship, safety, cost-effectiveness, and equity across multicenter prospective cohorts or randomized trials.

**Supplementary Information:**

The online version contains supplementary material available at https://doi.org/10.1186/s42836-026-00444-0.

## Introduction

Total knee arthroplasty (TKA) remains the definitive surgical treatment for end-stage knee osteoarthritis, offering reliable pain relief and functional improvement for appropriately selected patients [1, 2]. Despite the overall success of modern TKA, postoperative dissatisfaction remains a clinically important problem. Dissatisfaction is rarely attributable to a single mechanism; it may reflect implant positioning, soft-tissue imbalance, preoperative pain sensitization, unmet expectations, psychological factors, comorbidity burden, rehabilitation, and patient-specific anatomical variation [3–5].

Robotic-assisted and computer-assisted technologies were introduced to improve the reproducibility of bone preparation, implant positioning, limb alignment, and surgical planning [6–9]. Contemporary platforms, including Mako, ROSA, CORI/NAVIO, and related navigation-assisted systems, have demonstrated technical advantages over conventional manual instrumentation in several studies, particularly with respect to alignment accuracy and reduction of radiographic outliers [6–9]. These findings support the value of robotic and navigation technologies as precision-enhancing tools. However, improved technical precision does not automatically establish durable superiority in patient-reported outcomes, implant survivorship, or cost-effectiveness [8, 9].

The rapid expansion of AI has introduced a second layer of complexity. AI and machine learning (ML) may support radiographic interpretation, implant sizing, patient selection, dissatisfaction prediction, operative planning, intraoperative feedback, and postoperative monitoring [10–18]. These functions are not synonymous with robotic execution. A robotic system may assist bone preparation without using AI, whereas an AI model may predict dissatisfaction or implant size without being embedded in a robotic platform.

This distinction is central to the interpretation of the current literature. Terms such as robotic surgery, navigation-assisted surgery, digital planning, AI-assisted surgery, and ML prediction are frequently used inconsistently. As a result, claims about “AI-assisted robotic TKA” may inadvertently combine evidence from different technologies and different clinical questions. The purpose of this review is therefore not to claim comparative superiority of AI-enabled robotic TKA, but to clarify terminology, map the evidence landscape, and identify what remains unproven.

This contribution is distinct from existing reporting and appraisal frameworks. TRIPOD/TRIPOD-AI, PROBAST, and DECIDE-AI provide guidance for reporting or appraising an individual prediction model, and SANRA provides guidance for the conduct of a narrative review; none of these frameworks address the TKA-specific problem targeted here, namely that robotic-execution evidence and AI/ML prediction evidence are frequently cited interchangeably under the single label “AI-assisted robotic TKA.” Unlike prior AI/robotic TKA reviews, this manuscript organizes the evidence by pathway stage and clinical function (Tables 1 and 2) and keeps robotic-execution evidence and AI/ML evidence analytically separate at the level of each function, rather than treating AI-enabled robotic TKA as a single intervention.

**Table 1:** Operational definitions used in this review

Term	Definition	Robotic component?	AI/ML component?	Role in this review
Conventional manual TKA	TKA performed using standard instrumentation without robotic or computer-assisted execution	No	No	Baseline comparator
Computer-assisted or navigation-assisted TKA	Digital guidance for alignment or positioning, usually without robotic execution	Limited/variable	Not necessarily	Related precision technology
Robotic-assisted TKA	Robotic platform used for planning, bone preparation, haptic guidance, or execution assistance	Yes	Not necessarily	Robotic precision evidence
AI/ML prediction model	Algorithm used for diagnosis, implant sizing, patient selection, dissatisfaction prediction, outcome prediction, or risk stratification	No/variable	Yes	AI pathway evidence
AI-enabled intraoperative decision support	AI/ML explicitly integrated into the robotic or intraoperative workflow to support planning, adaptation, or decision-making	Yes/variable	Yes	Emerging target concept

**Table 2:** Evidence maturity and required next steps for AI-related functions in the TKA pathway

Clinical function	Most relevant evidence type	Direct robotic integration?	Current interpretation	Required next step
Robotic alignment precision	Robotic/navigation comparative studies	Yes, but often not AI-specific	**Mature **^**a**^**.** Technical precision evidence is relatively mature; AI-specific effect is often not separable. This label is a relative, function-specific classification restricted to technical/radiographic endpoints and does not imply patient-level or long-term clinical superiority	Direct comparisons separating robotic execution from AI functionality
Implant sizing prediction	AI/ML prediction and planning studies	Usually no	**Early-to-moderate.** Potential workflow and planning value; clinical utility remains early	Prospective workflow validation and surgeon override reporting
Dissatisfaction prediction	AI/ML outcome models	No	**Early-to-moderate.** Relevant for counselling and expectation management; not evidence of surgical effectiveness	External validation, calibration, and decision-impact studies
Patient selection and planning	Risk-stratification and prediction models	Variable	**Early.** Promising but heterogeneous and often retrospective	Multicenter validation with subgroup performance
Intraoperative adaptive decision support	Technical or pilot systems	Possible	**Early.** Conceptually important but clinically immature	Prospective multicenter comparative trials with safety reporting
Postoperative monitoring	Wearables, PROMs, and recovery prediction models	No/variable	**Early.** Early evidence; integration into clinical pathways remains limited	Studies linking monitoring to interventions and outcomes
Cost-effectiveness and equity	Economic and implementation studies	Variable	**Early.** Highly context-dependent; AI-specific costs often not isolated	ICER/QALY-based real-world evaluation and access analysis

^a^All maturity labels in this table (Mature, Early-to-moderate, Early) are relative, function-specific classifications derived from the evidence-maturity criteria defined in Sect. 2 (Review scope and evidence interpretation); they describe the technical/methodological maturity of evidence for a given clinical function and should not be read as absolute statements of clinical maturity or as claims of patient-level or long-term comparative superiority

The review is organized around three practical questions. First, which AI-related functions are currently being discussed or evaluated across the TKA pathway? Second, which claims are supported by robotic or AI-specific evidence, and which remain inferential? Third, what types of validation are needed before AI-enabled robotic TKA can be considered a clinically established intervention rather than an emerging pathway-level innovation?

### Review scope and evidence interpretation

This article is a critical narrative review and evidence map. It is not a pooled systematic review or meta-analysis. The available literature was assessed according to clinical function and evidentiary role rather than treated as a single homogeneous intervention. Peer-reviewed literature relevant to robotic-assisted TKA, navigation-assisted TKA, AI/ML prediction models, digital planning, implant sizing, intraoperative decision support, postoperative monitoring, implementation, and economic considerations was reviewed narratively.

Searches were conducted in PubMed, Embase, Web of Science, Google Scholar, and reference lists of relevant reviews for English-language peer-reviewed literature. Eligible literature was published between January 2015 and January 2025, a period selected because it captures the introduction and subsequent clinical expansion of the robotic and navigation platforms discussed in Sect. 3 (Conceptual evolution of technology around TKA) and the emergence of AI/ML prediction literature in orthopaedics; the last search was run on 15 April 2026. Search concepts included total knee arthroplasty, robotic-assisted surgery, navigation-assisted surgery, computer-assisted surgery, digital planning, artificial intelligence, machine learning, deep learning, prediction modeling, implant sizing, decision support, patient selection, postoperative monitoring, cost-effectiveness, and implementation. The aim was interpretive mapping and conceptual clarification; therefore, formal PRISMA-style screening counts, pooled effect estimation, and GRADE certainty ratings were not attempted. The full search string used for PubMed, including Boolean operators and MeSH terms, is provided as a supplementary file. Studies were included if they were peer-reviewed, English-language, and addressed a defined TKA-related robotic, navigation, or AI/ML function corresponding to a category in Table 1; studies were excluded if they were conference abstracts without an available full text, addressed hip or other joints only and were used for background rather than evidence, or could not be mapped to a Table 1 category. Each eligible study was assigned to a Table 1 category based on the explicit presence or absence of robotic execution and of an AI/ML component as reported by the study authors; studies addressing more than one function (for example, an AI-based sizing tool evaluated in a robotic cohort) were cross-listed rather than forced into a single category. A PRISMA-ScR-informed flow diagram summarizing records identified, screened, and included is provided as Supplementary Fig. S1. Consistent with PRISMA-ScR guidance, systematic reviews and meta-analyses (*n* = 5) were excluded from the flow diagram because they represent synthesis of other studies rather than primary evidence. However, per Sect. 2 (Review scope and evidence interpretation), these reviews were explicitly used for background, terminology clarification, and contextual evidence interpretation (Table 3: Interpretation boundaries).

**Table 3:** Interpretation boundaries for common evidence sources in AI-related TKA literature

Evidence source	What it can support	What it cannot support
Robotic TKA alignment study	Claims about technical precision, alignment, outliers, and execution reproducibility	Claims that AI itself improves long-term outcomes
Navigation-assisted TKA study	Claims about digital alignment guidance and related precision technologies	Claims about robotic execution or AI-enabled decision support
AI implant-sizing model	Claims about algorithmic sizing prediction or planning feasibility	Claims about improved patient-reported outcomes or survivorship
AI dissatisfaction model	Claims about risk prediction, counselling potential, or expectation management	Claims about surgical superiority of AI-enabled robotic TKA
Economic implementation study	Contextual claims about costs, resource use, and adoption barriers	Generalizable cost-effectiveness without ICER/QALY modelling
Narrative/systematic review	Background, terminology, citation chaining, and context	Primary clinical effectiveness unless based on direct comparative evidence

Screening and category assignment (Table 1) were performed by a single reviewer (M.A.M.); this narrative review did not employ independent, duplicate screening by a second reviewer. Resulting category assignments and evidence-maturity classifications (Table 2) were subsequently reviewed and approved by all co-authors as part of manuscript review and critical revision, but co-authors did not independently re-screen the literature or re-assign categories. This single-reviewer design, while consistent with common practice for narrative reviews, differs from the independent duplicate screening expected of systematic reviews and is acknowledged as a limitation in Sect. 8 (Limitations). The evidence-maturity tiers used in Table 2 were assigned using explicit, checkable features rather than narrative judgement alone: a function was classified as mature when supported by multiple independent, largely consistent studies with at least partial external validation; moderate when supported by several studies of mixed design with limited external validation; and early when the evidence was predominantly single-center, retrospective, or pilot-level.

The decision not to perform quantitative pooling was deliberate. The available studies do not consistently evaluate the same intervention, comparator, outcome, or validation target. Some studies address robotic execution accuracy, others examine navigation-assisted alignment, and others report AI-based prediction models that are not embedded in a robotic platform. Combining these studies into a single comparative estimate would obscure rather than clarify the evidence base.

Review articles, systematic reviews, and broad contextual sources were used for background, terminology clarification, and citation chaining. They were not treated as primary clinical evidence for the effectiveness of AI-enabled robotic TKA. Primary clinical studies, comparative robotic TKA studies, AI/ML prediction studies, implementation studies, and economic analyses were interpreted separately according to the clinical question they addressed. Studies resting on TKA-specific primary studies are flagged inline as direct evidence. Where a statement relies on broader orthopaedic or indirect evidence (e.g., hip arthroplasty data, general ML principles), this is explicitly noted to maintain transparency about evidence specificity.

Clear definitions are necessary because many studies do not explicitly separate robotic assistance from AI functionality. In this review, robotic-assisted TKA refers to robotic planning, haptic guidance, or execution assistance. AI-enabled intraoperative decision support is reserved for systems in which an AI or ML component is explicitly integrated into intraoperative planning, adaptation, or decision-making. The operational definitions used to distinguish between these technologies are summarized in Table 1. AI/ML prediction models are considered separately when they support diagnosis, implant sizing, patient selection, satisfaction prediction, risk stratification, or postoperative monitoring without being part of the robotic execution workflow.

Evidence was interpreted conservatively. Robotic or navigation-assisted TKA studies were used to inform claims about technical precision and alignment. AI/ML studies were used to inform claims about prediction, planning, risk stratification, and decision support. Studies of total joint arthroplasty or total hip arthroplasty were not used to infer TKA-specific effectiveness except as broad implementation context. This function-specific interpretation is intended to avoid the central methodological error of treating heterogeneous studies as if they evaluated one intervention.

### Conceptual evolution of technology around TKA

The technological evolution of TKA can be understood as a gradual shift from surgeon-executed manual instrumentation toward increasingly data-rich planning, precision execution, and pathway-level decision support. Conventional manual TKA remains the clinical benchmark, supported by decades of outcome data and registry experience. Computer navigation introduced digital alignment guidance, whereas robotic-assisted systems added more reproducible execution of planned bone resections and implant positioning.

AI does not simply represent the next version of the robot. It may operate before, during, or after the operation, and in many cases it is not physically connected to robotic execution. Preoperative AI may help interpret imaging, estimate implant size, stratify risk, or identify patients at higher risk of dissatisfaction. Intraoperative AI may eventually support adaptive planning, soft-tissue balancing, kinematic assessment, or computer-vision-assisted decision support. Postoperative AI may support recovery monitoring, complication prediction, rehabilitation optimization, and longitudinal assessment of patient-reported outcomes.

**Fig. 1:**
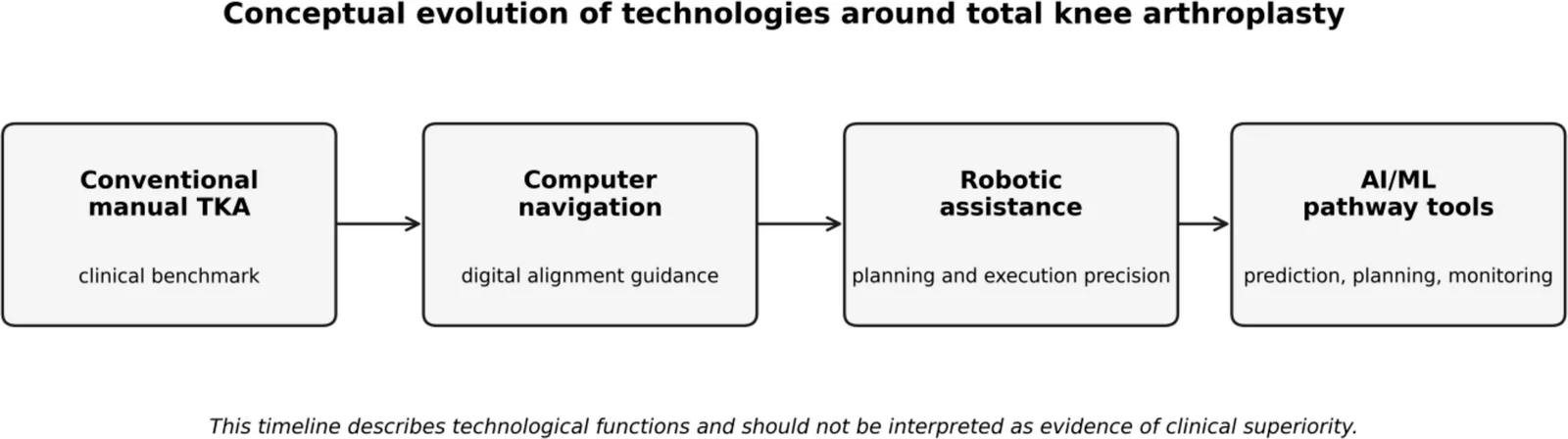
Conceptual evolution of technologies around total knee arthroplasty. The timeline shows technological functions and evidence boundaries rather than a hierarchy of proven clinical superiority. Conventional manual TKA remains the long-term clinical benchmark, while AI/ML tools represent emerging pathway-level functions that require function-specific validation

Figure 1 presents this evolution as a conceptual timeline. The figure should not be interpreted as evidence that later technologies are automatically superior. Rather, it illustrates why a single phrase such as “AI-assisted robotic TKA” is too broad unless the specific function, comparator, and outcome are defined.

### Evidence map across the TKA pathway

**Fig. 2:**
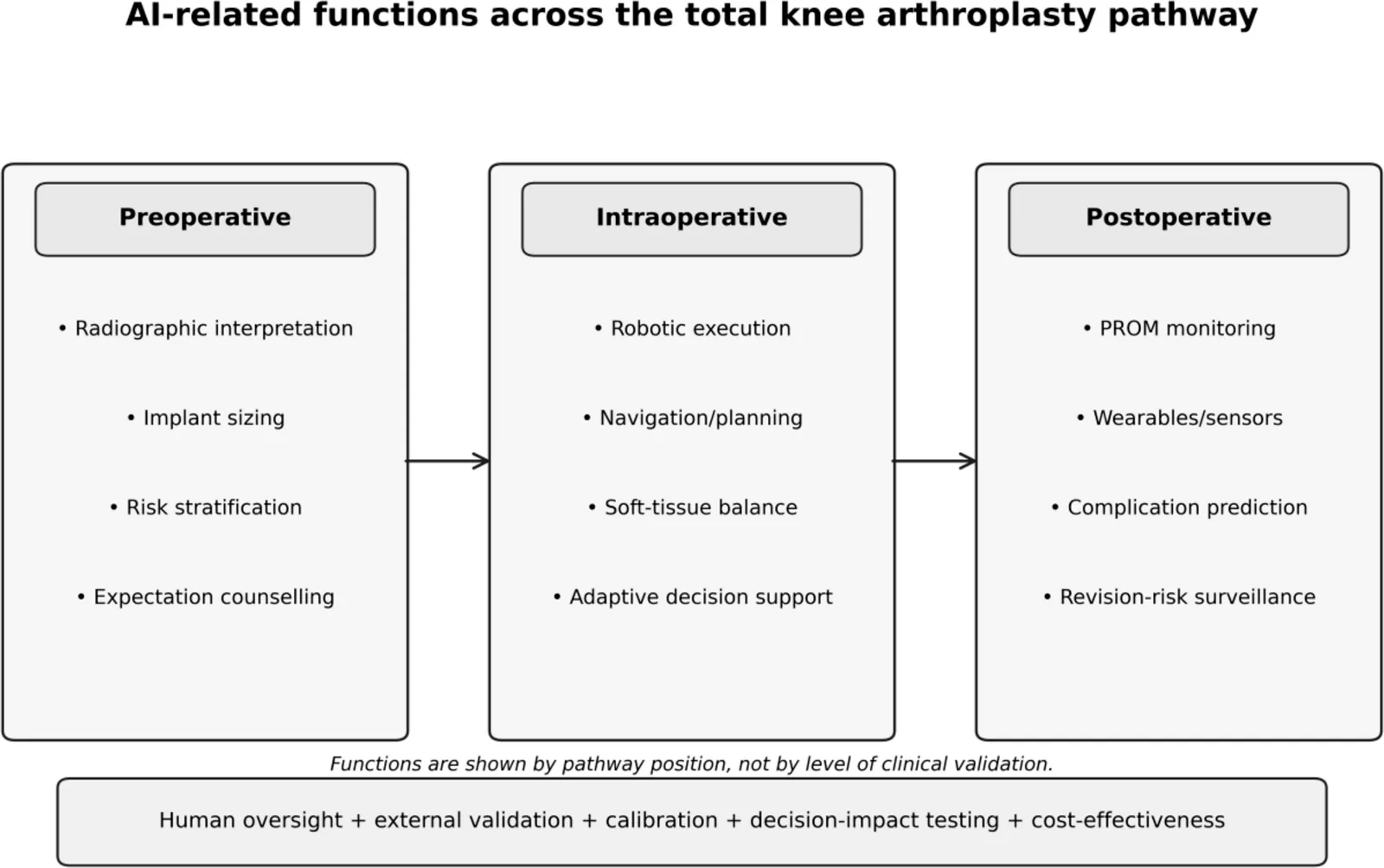
Conceptual framework of AI-related functions across the TKA pathway. Artificial intelligence may contribute to preoperative planning and risk stratification, intraoperative decision support, and postoperative monitoring. The figure is an author-generated conceptual schematic and should not be interpreted as evidence that AI-enabled robotic TKA has established clinical superiority over conventional robotic or manual TKA

Figure 2 provides a conceptual framework illustrating how AI functions are distributed across the preoperative, intraoperative, and postoperative phases of the TKA pathway.

### Robotic and navigation-assisted TKA

The strongest and most mature evidence for robotic-assisted and navigation-assisted TKA comes from TKA-specific comparative and cohort studies [19–23]. These systems may improve the reproducibility of implant positioning, reduce radiographic alignment outliers, and support more consistent execution of preoperative plans compared with conventional instrumentation. These findings support the technical value of robotics and navigation.

However, in many studies, the contribution of AI is either absent, unclear, or not separable from the robotic platform itself. Alignment improvements observed in robotic or navigation-assisted TKA should therefore not automatically be interpreted as proof that AI adds incremental benefit beyond robotic assistance. A more defensible interpretation is that robotic and navigation systems can improve technical precision, whereas the AI-specific contribution remains incompletely validated. Consistent with the evidence-source boundaries in Table 3, the robotic/navigation alignment studies cited in this section support claims about technical precision and alignment only; they do not, on their own, support claims about the incremental value of AI, which are addressed separately in Sect. 4.2 (AI/ML prediction and planning models).

This distinction matters clinically: alignment and component positioning are intermediate technical endpoints, not proof of durable patient-reported benefit. The gap between technical precision and patient-centered outcomes is developed in full in Sect. 4.4 (Patient-reported outcomes and the alignment-to-function gap) and Sect. 5 (Interpretation of the evidence).

### AI/ML prediction and planning models

AI/ML models have been applied to several TKA-relevant tasks outside the intraoperative robotic workflow. These include prediction of implant sizing, postoperative dissatisfaction, outcome trajectories, radiographic severity, length of stay, complications, and patient-selection variables [24–28]. Such models may eventually support individualized counselling, operative planning, expectation management, and perioperative risk stratification. Statistical prediction of many of these outcomes predates AI/ML methods; conventional regression-based models for dissatisfaction, complications, and length of stay have been reported for over a decade. The contribution of AI/ML in this domain is therefore primarily to scale, automate, and standardize access to prediction that was already methodologically possible, rather than to introduce an entirely new evidence base, and this framing is used throughout the remainder of this section.

Machine-learning models for dissatisfaction prediction are particularly relevant because postoperative satisfaction is influenced by factors that are not fully captured by alignment alone [3–5, 18, 25, 29, 30]. Selected TKA-specific studies using machine-learning and AI prediction models have reported moderate discrimination (AUC range 0.70–0.82) for dissatisfaction or satisfaction-related outcomes after TKA [18, 25, 29, 30]. However, these studies should be interpreted as evidence of AI-based prediction, not as evidence that AI-enabled robotic surgery improves outcomes. Considered critically, the cited dissatisfaction-prediction studies are predominantly retrospective, single-institution analyses with sample sizes ranging from several hundred to a few thousand patients; discrimination is generally reported as moderate (AUC in the range typically described as acceptable rather than excellent), and external validation in an independent cohort is reported in only a minority of these studies. Calibration and decision-impact analyses are rarely reported. This pattern, rather than a general assertion of weakness, is the basis for describing this evidence as insufficiently externally validated.

Implant-sizing and planning algorithms may support preoperative workflow and templating, particularly when paired with digital imaging and patient-specific planning. Their value should be judged by sizing accuracy, reduction in planning time, avoidance of intraoperative mismatch, effect on inventory management, and integration into clinical workflow. At present, many such models remain retrospective or technical, and prospective workflow studies are needed before claims of clinical utility can be made.

The most defensible conclusion is that AI/ML has potential as a decision-support layer around TKA, especially before and after surgery, subject to the external-validation limitations already noted above and developed further in Sect. 5 (Interpretation of the evidence). Discrimination alone is insufficient. Clinical deployment requires calibration, subgroup performance, decision-impact testing, clinician usability, and evidence that the model beneficially changes decisions.

### Intraoperative AI and adaptive decision support

True intraoperative AI-enabled robotic decision support remains at an earlier stage of clinical validation. Proposed functions include adaptive planning, real-time soft-tissue balancing assistance, kinematic feedback, computer-vision support, and dynamic adjustment of surgical plans according to patient-specific anatomy or intraoperative measurements [12, 13, 16, 17].

These applications are conceptually important because they may eventually distinguish AI-enabled surgery from conventional robotic execution. In theory, intraoperative AI could help translate static preoperative planning into a more dynamic model of joint balance, kinematics, and patient-specific anatomy. Such functionality could be clinically meaningful if it improves stability, range of motion, pain, satisfaction, or implant survivorship.

At present, however, most intraoperative AI evidence remains technical, pilot-level, or indirect. The few TKA-specific studies published are predominantly single-center proof-of-concept work. Broader orthopaedic literature and general AI principles inform theoretical discussions, but direct TKA-specific clinical validation of intraoperative adaptive AI decision support is notably limited. Robust multicenter comparative studies are needed before these systems can be considered clinically validated interventions. At a minimum, future intraoperative AI studies should report the input data, decision logic, level of automation, surgeon override process, failure modes, safety monitoring, and whether the AI component changed the surgical plan or outcome. The term “AI-assisted robotic TKA” should therefore be used cautiously and only when the AI component is explicitly described.

### Patient-reported outcomes and the alignment-to-function gap

A recurring issue in robotic TKA research is the gap between technical precision and patient-reported benefit. Improved alignment is valuable, but TKA satisfaction is multifactorial. This conclusion is supported by TKA-specific observational and cohort studies documenting patient-reported outcomes. Soft-tissue balance, pain mechanisms, preoperative function, expectations, psychological factors, rehabilitation, comorbidity burden, and implant-related factors all influence outcomes [3–5].

This gap explains why evidence of improved mechanical alignment cannot be interpreted as definitive evidence of improved patient experience. A patient may have technically accurate component placement and still experience dissatisfaction because of stiffness, instability, persistent pain, unrealistic expectations, depression, comorbidity burden, or poor rehabilitation. Conversely, many patients do well after conventional TKA despite less technologically intensive execution.

AI may help address this gap by supporting patient selection, expectation counselling, implant planning, and postoperative monitoring. These applications represent emerging directions with promising theoretical frameworks; as noted in Sect. 4.2 (AI/ML prediction and planning models), the current evidence remains limited to single-center or retrospective work with minimal external validation. These applications are attractive precisely because they target variables that robotic precision alone cannot address. However, their value requires prospective decision-impact studies rather than retrospective performance reporting alone. The key question is not only whether a model predicts an outcome, but whether using the model improves counselling, shared decision-making, perioperative planning, or patient-centered outcomes.

### Postoperative monitoring and longitudinal care

Postoperative AI applications may include wearable-sensor analysis, remote monitoring, patient-reported outcome tracking, early detection of complications, rehabilitation optimization, and prediction of recovery trajectories [12, 13, 17]. These sources are broader orthopaedic and digital-health literature rather than TKA-specific postoperative-monitoring studies; they are cited here for indirect, conceptual support, and TKA-specific direct evidence for postoperative AI monitoring remains limited. These applications fit naturally into a longitudinal care model because TKA outcomes evolve over months to years and are influenced by rehabilitation, adherence, complications, and patient expectations.

The postoperative setting may be particularly suitable for AI because data can be collected repeatedly and dynamically. Rather than relying only on clinic visits, remote tools may provide information about mobility, pain trajectories, function, sleep, activity level, and recovery deviation. Such systems could support earlier intervention when recovery is delayed, but only if the signals are reliable, interpretable, and integrated into clinical workflows.

Postoperative AI remains less developed than preoperative prediction or robotic alignment technology. The current evidence base for postoperative AI in TKA consists primarily of single-center, retrospective, or pilot studies. Prospective multicenter validation with decision-impact and outcome-linkage studies is needed before deployment in routine clinical practice. Future studies should determine whether postoperative monitoring systems improve clinically meaningful outcomes, such as functional recovery, complication detection, readmission, revision risk, patient satisfaction, and healthcare utilization. Validation should include not only predictive accuracy but also clinician response pathways and patient acceptability.

### Cost, implementation, and equity

Robotic and AI-related technologies introduce costs related to capital acquisition, software licensing, disposable instrumentation, staff training, operating-room workflow, maintenance, and updates [12, 13, 23, 31, 32]. Of these, only [23] reports direct TKA-specific cost/length-of-stay data; [12, 13] are broader orthopaedic digitalization literature, and [31, 32] report length-of-stay and readmission data for total joint arthroplasty and total hip arthroplasty, respectively, and are used here only as indirect implementation context rather than TKA-specific economic evidence. Reported costs are highly context-dependent and cannot be generalized across healthcare systems without formal economic evaluation. The current evidence maturity and required next steps for these diverse AI-related functions are summarized in Table 2.

The relevant question is not whether AI-assisted TKA is technologically impressive, but whether it improves outcomes enough to justify its incremental cost and implementation burden. Cost-effectiveness studies should include capital investment, case volume, learning curve, operating time, length of stay, readmission, revision risk, patient-reported outcomes, and quality-adjusted life years. Studies should also distinguish costs attributable to the robotic platform from costs attributable to AI software or decision-support functionality.

Equity should be considered from the outset. High-cost technologies may concentrate in large tertiary centers and high-income settings, limiting access for lower-resource systems. If AI models are trained primarily in high-volume centers using selected patient populations, their performance may not generalize to community hospitals, rural settings, or patients with complex comorbidity profiles. Implementation studies should therefore evaluate not only average performance but also subgroup performance, access, and fairness.

### Interpretation of the evidence

The central finding of this review is that AI-related evidence around robotic TKA is distributed across several related but non-equivalent functions. Robotic-assisted TKA has comparatively stronger evidence for improving technical precision. AI/ML evidence is most developed in prediction and planning tasks adjacent to surgery. However, as detailed in Sect. 4.4 (Patient-reported outcomes and the alignment-to-function gap), direct evidence that AI-enabled robotic TKA improves long-term patient-reported clinical outcomes remains limited. This gap between technical precision and patient-centered benefit is the primary reason that current claims of AI-enabled superiority remain premature.

This distinction is more than semantic. A technically successful robotic alignment study cannot prove the value of AI. Similarly, a machine-learning model that predicts dissatisfaction cannot prove the effectiveness of AI-enabled intraoperative decision support. Evidence should therefore be interpreted according to the specific clinical function being evaluated.

The field should move from broad claims of “AI-assisted robotic superiority” toward function-specific evaluation. Implant-sizing algorithms should be evaluated according to sizing accuracy, operative planning efficiency, and workflow impact. Dissatisfaction prediction models should be evaluated according to discrimination, calibration, external validation, decision impact, and patient communication outcomes. Intraoperative adaptive systems should be evaluated according to safety, reproducibility, surgeon interaction, soft-tissue balance, operative time, functional outcomes, and long-term survivorship. Economic analyses should be anchored in real-world implementation costs and patient-centered benefits.

This function-specific approach also helps authors avoid overstatement. The most defensible current conclusion is not that AI-assisted robotic TKA is clinically superior, but that AI-related tools are emerging around TKA in several clinically plausible roles. Some roles, such as robotic precision and radiographic assessment, are supported by more mature evidence. Others, such as intraoperative adaptive AI and cost-effectiveness, remain early and require prospective validation. To guide the reader, the appropriate interpretation boundaries for common evidence sources in this literature are provided in Table 3.

### Proposed function-specific evaluation framework

Building on the function-specific interpretation established in Sect. 5 (Interpretation of the evidence), a function-specific evaluation framework should define, for each AI-related TKA technology, the clinical decision being supported, the intended user, the comparator, the outcome, and the validation standard.

For preoperative prediction models, essential reporting elements include input variables, missing-data handling, discrimination, calibration, external validation, subgroup performance, and evidence of clinical utility. For implant-sizing or planning models, studies should report sizing accuracy, concordance with intraoperative decisions, workflow effect, revision of planned component size, and surgeon override. For intraoperative systems, studies should report data inputs, level of automation, surgeon control, safety stops, failure modes, and the specific decision changed by the algorithm.

### Reporting recommendations for future studies

Consistent with the terminology distinction established in Sect. 5 (Interpretation of the evidence), future studies should avoid using “AI-assisted robotic TKA” as an undefined broad label; the intervention should be described with sufficient detail to allow replication and interpretation. Essential information includes whether the AI component is used before, during, or after surgery; whether it is embedded in a robotic platform; what data it uses; what decision it supports; whether the surgeon can override the recommendation; and how performance is monitored after deployment.

Comparator selection is equally important. If the goal is to evaluate robotic precision, conventional manual TKA may be an appropriate comparator. If the goal is to evaluate AI-specific value, the comparator should ideally be non-AI robotic TKA or standard digital planning without AI. If the goal is to evaluate a prediction model, the comparator may be standard clinical judgement, a conventional statistical model, or existing risk scores. Mixing these comparator structures within a single effectiveness claim should be avoided.

Outcome selection should match the function being evaluated. Alignment accuracy may be appropriate for robotic execution studies but is insufficient for patient-level AI claims. Prediction models should report calibration and decision impact, not only AUC. Intraoperative AI should report safety and failure modes, not only technical feasibility. Economic studies should include cost-effectiveness and opportunity cost, not only procedure-level pricing.

Finally, AI studies should include diversity-aware validation. Performance should be tested across age, sex, body mass index, ethnicity, comorbidity burden, deformity type, implant platform, surgeon experience, center volume, and healthcare setting where possible. Without such validation, algorithmic performance may appear strong in development cohorts while failing in real-world deployment. A summary of minimum reporting recommendations for future studies is presented in Table 4.

**Table 4:** Minimum reporting recommendations for future AI-related TKA studies

Reporting item	Minimum information needed
AI component	Define the algorithm, input data, output, and intended decision
Timing in pathway	Specify whether the tool is preoperative, intraoperative, postoperative, or longitudinal
Relationship to robot	State whether AI is embedded in the robotic platform or separate from robotic execution
Comparator	Use manual TKA, navigation, non-AI robotic TKA, clinician judgement, or statistical models as appropriate
Validation	Report internal validation, external validation, calibration, subgroup performance, and failure modes
Clinical utility	Assess decision impact, workflow effect, patient outcomes, and clinician usability
Safety and governance	Describe oversight, surgeon override, monitoring, and post-deployment quality control
Economic evaluation	Include capital costs, licensing, disposables, training, time, revisions, readmissions, and QALYs

### Limitations

This review has several limitations. First, it is a critical narrative review and evidence map rather than a formal systematic review or meta-analysis. The conclusions are therefore interpretive and should not be read as pooled comparative-effectiveness estimates. Second, the literature is conceptually heterogeneous, and many studies do not clearly separate robotic assistance, navigation, digital planning, AI prediction, and intraoperative decision support. Relatedly, literature identification and screening were performed by a single reviewer rather than by independent duplicate screening; this is a recognized limitation of single-reviewer narrative reviews and may increase the risk of selection or classification error compared with a team-based systematic review. As noted in Sect. 2 (Review scope and evidence interpretation), resulting category assignments were reviewed and approved by all co-authors, which partially mitigates but does not eliminate this risk.

Third, many AI/ML studies are retrospective and lack external validation. Fourth, long-term outcomes such as implant survivorship, revision risk, and durable patient-reported benefit remain insufficiently studied. Fifth, economic evidence remains limited and highly context-dependent. Sixth, rapidly evolving technology means that terminology and evidence maturity may change quickly, requiring periodic reassessment of the literature.

Despite these limitations, the evidence-map approach is appropriate for the present state of the field because it prevents premature pooling of non-equivalent interventions. By separating robotic precision, AI prediction, digital planning, intraoperative adaptation, postoperative monitoring, and implementation evidence, this review provides a more cautious and interpretable framework for future research.

### Future directions

The specific reporting elements required for AI prediction models, intraoperative AI systems, and economic evaluations are detailed in Sect.  7 (Reporting recommendations for future studies) and Table 4 and are not repeated here; the priority for the next phase of research is applying that checklist prospectively, in multicenter cohorts, with the appropriate comparator for each function. Future trials should also examine whether AI-supported planning or counselling changes patient expectations, shared decision-making, and rehabilitation engagement. These outcomes are important because patient satisfaction after TKA is not determined by technical alignment alone. The most valuable AI tools may ultimately be those that integrate technical precision with individualized risk communication and longitudinal care.

## Conclusion

Robotic-assisted TKA is best supported as a precision-enhancing technology, particularly for improving the reproducibility of alignment and implant positioning. AI in the TKA pathway should presently be understood not as a single validated surgical intervention but as a group of emerging functions that may support imaging interpretation, implant sizing, patient selection, dissatisfaction prediction, planning, intraoperative decision support, and postoperative monitoring.

The incremental clinical value of AI beyond conventional robotic assistance remains insufficiently established. Future work should avoid broad claims of AI-assisted superiority and instead pursue transparent, function-specific, externally validated, and patient-centered evaluation. This distinction is essential for scientific clarity, responsible translation, and fair assessment of whether AI meaningfully improves outcomes in total knee arthroplasty. The contribution of this review is not a new reporting checklist, a role already served by TRIPOD/TRIPOD-AI, PROBAST, DECIDE-AI, and SANRA, but a TKA-specific evidence map that keeps robotic-execution evidence and AI/ML evidence analytically separate at the level of each clinical function, and that identifies where this separation is not currently respected in the literature.

## Supplementary Information

Supplementary Material 1.

## Data Availability

No datasets were generated or analysed during the current study.

## Abbreviations

AI: Artificial intelligence
AUC: Area under the receiver operating characteristic curve
BMI: Body mass index
CNN: Convolutional neural network
CORI: CORI surgical system
CT: Computed tomography
HKA: Hip-knee-ankle angle
ICER: Incremental cost-effectiveness ratio
KOA: Knee osteoarthritis
KSS: Knee Society Score
LOS: Length of stay
ML: Machine learning
NAVIO: NAVIO surgical system
OA: Osteoarthritis
OKS: Oxford Knee Score
PROM: Patient-reported outcome measure
QALY: Quality-adjusted life year
RCT: Randomized controlled trial
ROSA: Robotic Surgical Assistant
SaMD: Software as a Medical Device
THA: Total hip arthroplasty
TJA: Total joint arthroplasty
TKA: Total knee arthroplasty
