# Supplementary material for: Distinguishing artificial intelligence from robotic execution in total knee arthroplasty: a narrative review and evidence map
Source: Arthroplasty. 2026 Nov 6;8:86. doi: 10.1186/s42836-026-00444-0 (PMC13741776; doi:10.1186/s42836-026-00444-0)
Supplement: Supplementary file 1 [file 42836_2026_444_MOESM1_ESM.docx]

**Supplementary Fig. S1.** PRISMA-ScR-informed flow diagram of the literature identification and selection process for the narrative review.

**Figure Legend:**

**1. Primary Evidence Base (*n* = 24):**

These 24 studies represent the primary evidence base evaluated in the narrative review (Sections 4.1–4.5 of the main text, Evidence map across the TKA pathway). They address AI/ML-specific functions, robotic-assisted TKA execution, or both. These are the studies upon which the evidence-maturity classifications (Table 2) are based, and they are the only studies tracked in the PRISMA-ScR flow diagram.

**2. Total References Cited (*n* = 32):**

The manuscript cites 32 references: 24 primary evidence-based studies and 8 supplementary sources. The 24 primary studies address AI/ML-specific functions, robotic-assisted TKA execution, or both, and are the only studies tracked in the PRISMA-ScR flow diagram. The 8 supplementary sources are used for background, terminology clarification, and contextual evidence interpretation and are not treated as primary evidence. They comprise 5 systematic reviews or meta-analyses and 3 foundational contextual studies. In total: 24 + 8 = 32 references.

**3. Systematic Reviews and Meta-Analyses (*n* = 5):**

These 5 systematic reviews and meta-analyses were retrieved during the search and used for background, terminology clarification, and contextual evidence interpretation (Table 3: Interpretation boundaries). They are not included in the primary evidence base and are not tracked in the PRISMA-ScR flow diagram because they represent synthesis of other studies rather than primary evidence. This distinction is consistent with PRISMA-ScR guidance, which tracks primary studies in the flow diagram while permitting reviews to be used for background and citation chaining. The flow diagram therefore tracks the primary evidence base only (*n* = 24), while the main text appropriately uses secondary literature for context.

**4. Screening Process:**

Records were identified through comprehensive searches of PubMed, Embase, Web of Science, and Google Scholar. After removal of duplicates and records removed for other reasons, records were screened at the title/abstract level. Records clearly outside the scope were excluded. The remaining records underwent full-text review for eligibility based on the Table 1 category mapping. Records were excluded at the full-text stage for the following reasons: lack of required information, different interventions, and inability to map to a specific TKA pathway function. The final 24 studies form the primary evidence base.

Source: ^[1]^

This work is licensed under CC BY 4.0. To view a copy of this license, visit <https://creativecommons.org/licenses/by/4.0/>

1.	Page MJ, McKenzie JE, Bossuyt PM, Boutron I, Hoffmann TC, Mulrow CD, et al. The PRISMA 2020 statement: an updated guideline for reporting systematic reviews. BMJ. 2021;372:n71.
